# Potential Use of AI Chatbots by Patients With Bowel Tumors: Opportunities and Implications for Low- and Middle-Income Countries

**DOI:** 10.7759/cureus.116502

**Published:** 2026-09-18

**Authors:** Jehad F AlSamhori, Abdel Rahman F AlSamhori, Rama M Amourah, Hazim Aldweik, Ahmad F AlSamhori, Leen M Holy, Rihane Zakraoui, Sawsan H Alomari, Awni D Shahait

**Affiliations:** 1 School of Medicine, The University of Jordan, Amman, JOR; 2 Department of Surgery, Southern Illinois University School of Medicine, Springfield, USA

**Keywords:** artificial intelligence (ai), bowel tumors, chatbots, colorectal cancer (crc), low- and middle-income countries (lmics)

## Abstract

Bowel tumors and colorectal cancer (CRC), in particular, have become a major concern to the global health community and have led to high morbidity and mortality levels, especially in lower- and middle-income countries (LMICs). This narrative review examines the potential roles of patient-facing AI chatbots and large language model (LLM)-based conversational tools across bowel tumor care, with particular attention to their potential applicability, limitations, and implementation requirements in LMICs. The available evidence spans small prospective and feasibility studies of patient-facing symptom monitoring or education as well as studies evaluating LLM performance in simulated clinical scenarios and clinician-facing decision support. Among the 16 studies, three were conducted in middle-income countries, and none in low-income countries; most evidence therefore arose from high-income settings. Patient-facing interventions generally support feasibility, acceptability, symptom reporting, and communication, whereas accuracy and concordance for screening, diagnosis, or treatment recommendations vary by task and are less reliable in complex cases. These findings support chatbots primarily as adjuncts to structured clinical care rather than autonomous clinical decision-making tools. AI chatbots may complement bowel tumor care through education, symptom monitoring, communication, and selected decision-support functions, but current evidence does not establish broad clinical effectiveness or safety, particularly in LMICs. Prospective validation, human oversight, privacy safeguards, local-language and cultural adaptation, digital-access considerations, and context-specific implementation studies are needed before wider clinical adoption.

## Introduction and background

Bowel tumors are an important concern due to their prevalence worldwide and their potential to progress into malignancies. These include several types of cancers, most notably colorectal cancer (CRC), which accounts for 9.6% of all cancers globally and is the second leading cause of cancer deaths [1,2]. According to the 2023 Global Burden of Disease (GBD) results, CRC accounted for about 1.1 million deaths out of 10.4 million all cancer-related deaths, representing 10.6%. In low- and middle-income countries (LMICs), CRC mortality reached 65,824, which is 5.9% of global CRC deaths [3,4]. Regarding disability-adjusted life years (DALYs), CRC contributed approximately 26.2 million DALYs globally, which corresponds to 9.6% of all cancer-related DALYs. In LMICs, CRC DALYs were around 1.9 million, representing 7.3% of the global DALY burden of this disease. These tumors are widespread across many populations for multiple reasons, which implies the importance of early diagnosis and treatment [3,4].

Given the prevalence of bowel tumors and despite the availability and diversity of technological advancements, there are significant disparities. Many LMICs lack access to or even basic knowledge of modern technology, due to significant limitations such as geographical infrastructure, gaps in resource distribution, and lack of experience and qualified physicians [5]. Razzano et al. reported that many countries have only one pathologist per million people. Somalia, for example, had none at that time, which highlights the need to integrate additional supportive facilities [6]. However, the key challenge is maintaining consistency; implementing innovations is not enough; training medical and non-medical staff allows the process to keep running steadily [7].

Artificial intelligence (AI) encompasses a broad range of computational approaches, including predictive models, computer vision, machine learning (ML), and generative systems. An AI chatbot is defined as a conversational interface that uses rule-based logic, ML methods, or a large language model (LLM) to interact with users through natural-language text or multimodal inputs. This distinction is important because imaging classifiers, radiogenomic models, and other non-conversational AI systems perform different clinical functions and should not be interpreted as direct evidence of chatbot effectiveness [8-10]. In oncology, chatbots are best considered tools that may complement clinical teams, with appropriate privacy, safety, and human-oversight safeguards.

AI chatbots have shown promising applications in cancer care, yet their role in bowel tumor care remains heterogeneous and incompletely defined. This article presents a narrative review for the potential use of AI chatbots by patients with bowel tumors. It synthesizes bowel tumor-specific studies together with selected evidence from broader gastrointestinal and oncology settings when relevant to chatbot implementation. Evidence from high-income settings is discussed for its potential transferability to LMICs and is not presented as direct evidence of effectiveness in LMIC populations. Using the current World Bank income classification, only three of the 16 studies were conducted in middle-income countries (Brazil, Argentina, and China), and none were conducted in low-income countries [11]. Because the included studies differ substantially in design, clinical role, comparator, model type, and outcome definition, findings are interpreted descriptively rather than as directly comparable effect estimates. This heterogeneity is also reflected in a recent 2026 systematic review of 37 LLM studies across CRC care, which used narrative rather than quantitative synthesis because of substantial differences in clinical tasks and outcomes [12].

## Review

AI chatbots in oncology

Chatbots are primarily classified as rule-based chatbots, ML chatbots, and LLM chatbots. Rule-based chatbots are suitable for simple symptom assessment and frequently asked questions, while ML/LLM chatbots give more personalized responses and psychosocial support [13,14].

In oncology, AI chatbots can support patients by answering questions about their diagnosis, treatment side effects, and lifestyle modifications. However, their understanding is limited, and patients may over-rely on chatbot responses for cancer-related decisions, especially when the chatbot communicates in ways that feel empathetic, which highlights the importance of physician supervision and clear guidance [13,15,16].

The development of LLM-based chatbots has expanded the potential applications of conversational AI in oncology beyond information retrieval. These systems can interpret natural-language questions, summarize medical information, explain aspects of cancer care in patient-oriented language, and provide responses to questions concerning treatment and supportive care. However, their clinical utility depends on the accuracy, completeness, consistency, and appropriateness of the generated responses. Consequently, evaluation should consider not only factual accuracy but also clinical relevance, safety, guideline concordance, and the potential for harmful or misleading recommendations [13,17].

Importantly, conversational AI chatbots should be distinguished from other AI/ML technologies used in oncology. Non-conversational AI diagnostic and imaging-based systems, including algorithms for lesion classification, segmentation, tumor detection, mammographic interpretation, digital pathology, and radiologic assessment, represent separate applications of AI and should not be considered evidence of chatbot effectiveness [17-26]. Maintaining this distinction is important because performance in a diagnostic or image-analysis task does not establish the effectiveness, safety, or clinical utility of a conversational chatbot.

Overall, AI chatbots represent a potentially useful adjunct for patient education, communication, and supportive care in oncology, but their role remains constrained by limitations in reliability, clinical contextualization, privacy, bias, and the risk of inappropriate reliance. Their implementation therefore requires appropriate clinical oversight, transparent communication regarding their limitations, and evaluation within the specific clinical and patient-facing context in which they are intended to be used [13,15,16].

Applications for bowel tumor patients

Applications

LLM-based chatbots have been progressing in terms of power, accuracy, versatility, and pervasiveness at an unprecedented rate [27,28]. There is a vast and growing undertaking of medical research to evaluate and implement chatbots and other LLMs in patient education, communication, screening, diagnosis, management, and follow-up [29-34]. Given the high and growing burden of bowel tumors on healthcare systems, the rise in technological availability and literacy, and the pressing need to address overtaxed healthcare professionals and systems, the question of chatbots’ role in the care of patients in need of bowel tumor screening, surveillance, and treatment is a pertinent one [35-37].

Symptom Assessment

Assessment of symptoms and side effects is one of the first and essential steps in establishing the diagnosis, determining the course of management, and understanding the impact of disease and its treatment on patient health and quality of life [38,39]. Chatbots have been reported to support symptom assessment and communication in patients with CRC who have used and interacted with these systems [40]. The technology’s accessibility and interactive features may facilitate timely reporting of symptoms and treatment-related issues, and features that support conversations may increase patient engagement with the technology [41,42]. In addition, carers and family members have used chatbots to report symptoms and side effects of patients receiving cancer treatment [43].

Therefore, chatbot-supported communication may offer a more comprehensive and timely manner to communicate patient-reported symptoms and treatment-related problems to healthcare providers [44]. However, there is currently no evidence that the use of chatbots alone results in increased diagnostic accuracy or enables individualised clinical management. Instead, chatbots may be adjuncts for symptom monitoring and communication with reported benefits depending on the clinical context and level of health care-professional involvement. Similarly, patient education and communication supported by a chatbot may also facilitate interactions with healthcare systems through increased patient engagement and participation in care [45]. Moreover, chatbots can offer carers an alternative way to document and communicate symptoms, which may help their role in advocating for patients with bowel tumors [46].

In a retrospective gynecologic oncology cohort, a clinician-supervised chatbot pathway was associated with fewer emergency department visits (adjusted incidence rate ratio (aIRR) 0.27, 95% CI 0.11-0.65) and unscheduled hospitalizations (aIRR 0.31, 95% CI 0.11-0.88); however, the study was small, non-randomized, and incorporated active monitoring by a cancer manager [37]. Taken together, the available evidence supports chatbots primarily as adjuncts to structured oncology care that may facilitate symptom capture, engagement, and escalation to clinicians. These findings should not be interpreted as evidence that chatbots independently optimize diagnosis or management.

Risk Stratification

AI has demonstrated accuracy, efficiency, and a clinically significant positive impact on risk stratification in medicine [47,48]. This is possible because of the ML techniques of feature selection and feature extraction. Feature selection is used to identify subsets of data that can be grouped as having the same attribute, or “feature,” whereas feature extraction is used to combine and transform an existing set of features into a dimensionally smaller set of features that are more useful for pattern identification and prediction [49,50]. However, most bowel-tumor evidence summarized here involves LLMs or multimodal models evaluated using constructed clinical scenarios, guideline prompts, endoscopic images, or other clinician-facing tasks rather than autonomous patient-facing risk assessment [51-56]. For example, ChatGPT-4o differentiated polypoid from non-polypoid colorectal lesions with 73% accuracy, comparable to expert and non-expert endoscopists for that specific task, but performed less well for more subtle morphological distinctions [34]. Such results are useful as proof-of-concept but do not establish real-world clinical effectiveness or safety. Chatbots have yet to reach the threshold of accuracy for clinical use, and are more dependent on the quality and quantity of data used for training and prompts than humans [57]. Further research and testing with larger and more diverse datasets may hone this technology to become suitable for use in healthcare [58]. Chatbots and other M-LLMs would be very useful for verifying the accuracy of physician findings and establishing consensus for these findings and their most appropriate management plan [59]. Thus, patient-facing chatbots may reasonably support collection of risk-factor information, education, and routing toward validated screening pathways, whereas definitive malignancy risk classification and management decisions should remain clinician-supervised. Larger prospective and externally validated studies are required before these tools can be used for independent risk stratification.

Pre- and Post-operative Guidance

Pre- and post-operative guidance and medicine require much work and coordination between patients and their healthcare providers with respect to patient education, assessment, and health optimization by modifying lifestyle, behaviors, and medications before and after surgery to reduce patient morbidity and mortality [60]. Accomplishing this goal while minimizing patient regret is achieved by prioritizing communication with and empowerment of patients to direct their perioperative care by making informed decisions [61,62]. Chatbots have been tested in multiple aspects of pre- and post-operative medicine, with promising results. Patients and healthcare professionals alike have expressed satisfaction with the medical advice and support provided by chatbots, being considered comparable or even superior to evidence-based sources with respect to addressing patient concerns [63,64]. Chatbots with image generation capabilities have also been explored for their potential to visually supplement patient education and communication [65]. Further refinement is required for chatbots to become accurate enough to be accepted for clinical use [52]; however, the accessibility, empathy, and flexibility of chatbots have great potential to facilitate communication and engage patients [66,67], and should not be ignored.

Healthcare professionals also stand to benefit from the integration of chatbots with perioperative guidance and care. Publicly available LLMs have demonstrated partial-to-high concordance with multidisciplinary team (MDT) recommendations in retrospective or simulated scenarios [68]. In one simulated preoperative assessment study, LLMs outperformed human participants for specific tasks such as surgical-fitness assessment, whereas overall secondary-outcome performance was comparable and human evaluators performed better for medication instructions [69]. These findings should therefore be interpreted as task-specific and hypothesis-generating rather than evidence that chatbots broadly surpass clinicians. Real-world implementation should retain clinician oversight, and further studies should evaluate feasibility, safety, workflow integration, and documentation benefits in actual clinical settings [59,70,71].

Chemotherapy Support

Some of the many challenges faced by chemotherapy patients include managing and reporting side effects, medication adherence, maintaining quality of life, and remaining proactive in their own lives and health [72]. Digital health interventions (DHIs) have been recognized as contributing to more timely, personalized, and effective management for chemotherapy patients for some time [73]. It therefore stands to reason that as chatbots become increasingly ubiquitous in digital technology, their impact on chemotherapy support is worth studying [74].

Chatbots may support chemotherapy care through reminders, symptom check-ins, education, and escalation pathways [42,75]. In the PENNY-GI intervention, the chatbot understood patient messages with 98.0% accuracy, but incorrect medication-management recommendations occurred in 7.3% of medication-related exchanges and incorrect symptom-management recommendations in 2.5% of symptom-related exchanges [75]. Gomaa et al. evaluated a hybrid text-messaging/chatbot intervention and demonstrated feasibility and acceptability, but overall psychosocial improvements were limited, and chatbot use itself was not associated with a significant change in patient activation [42]. Broader DHIs may improve engagement and self-management in some settings, but these effects should not be attributed solely to the chatbot component [33,76].

Survivorship Care

Understanding and implementing effective survivorship care is increasingly important as cancer survival improves [77,78]. AI has been explored for symptom monitoring and prediction in cancer survivors, although chatbots represent only one subset of this broader literature [79]. Conversational tools may provide accessible education, answer common questions, and support surveillance-related communication [63,80]. Studies using structured clinical scenarios have also reported variable guideline concordance for screening, surveillance, and treatment recommendations [56,68]. These findings demonstrate technical potential rather than established clinical effectiveness; complex or high-risk decisions remain particularly dependent on clinical context and human review. Accordingly, chatbots are best viewed as prospective adjuncts to survivorship care rather than autonomous decision-making systems [81-83].

Implications for LMICs

Resource Shortages

LMICs face substantial barriers to equitable cancer care, including shortages of healthcare workers and specialists, financial constraints, limited access to screening and diagnostics, and reduced availability of oncology infrastructure in rural areas [84-86]. DHIs may improve access and reduce travel burden in some contexts [87]. AI chatbots add conversational, multilingual, and potentially multimodal functions that could support patient education, symptom communication, and selected low-complexity tasks [65,80,88,89]. However, most supporting evidence currently comes from high-income settings, and these anticipated benefits should not be assumed to translate directly to LMIC health systems. Context-specific studies are needed to determine whether chatbots can improve access, outcomes, or workforce efficiency without worsening existing inequities [90,91].

Cost-Effectiveness Potential

Another major concern for LMICs is the high cost of cancer care, including both direct medical costs and nonmedical costs related to travel, accommodation, home care, disability, and lost productivity [92]. Potential mechanisms by which chatbots could reduce resource use include decreasing documentation burden, facilitating remote communication, and supporting timely triage or clinical decision-making [66,70,74]. Whether these mechanisms translate into net cost savings is not established. Formal economic evaluations should incorporate infrastructure, connectivity, integration, training, maintenance, governance, and model-use costs, as well as potential environmental impacts [28,54,93].

Geographic and Transportation Barriers

Rural bowel cancer patients in LMICs may face substantial travel burdens when specialized oncologic care is concentrated in tertiary centers [92,94]. Chatbots could facilitate remote education, symptom monitoring, and communication and may reduce some avoidable travel when integrated into clinician-supervised care pathways [33,36,40,43]. However, direct evidence that chatbot deployment reduces advanced disease, transportation costs, or adverse outcomes in LMIC populations remains limited and should be evaluated prospectively.

Language and Cultural Adaptation

Cultural and linguistic barriers can contribute to delayed or incorrect diagnosis, distress, and reduced trust between patients and healthcare professionals [95]. Translation technologies and conversational tools may improve accessibility, but chatbot performance can vary by language and may reproduce biases in training data, particularly for lower-resource languages and local practices [41,65,96-99]. Multilingual evaluations in colorectal and other clinical settings show that performance is not uniform across languages or countries [51,80,100-102]. Accordingly, local-language validation, culturally appropriate prompting, and human review are essential before deployment. Chatbots may help bridge selected communication gaps, but they should complement rather than replace qualified interpreters and clinical teams when nuanced communication is required [96,97,103].

Digital Literacy Challenges

Further research and integration of chatbots for clinical use must take into account the technological literacy of both healthcare professionals and patients. Knowledge and experience with chatbots is suboptimal among both healthcare practitioners and patients [43,64,80]. This barrier disproportionately affects LMICs [87]. Implementation of AI and chatbots without sufficient education and preparation of the population intended to use them can result in confusion, misunderstanding, and worse outcomes for both healthcare providers and patients than the baseline before the implementation of AI [104-106]. It is therefore imperative to address the gaps in AI and chatbot literacy, whether it be through the integration of AI experts in MDTs, modification of chatbots to produce outputs in simpler language, or increased initiatives to educate healthcare professionals about chatbots and increase their knowledge and familiarity with them [105,107,108].

Misinformation Risks

Given the growing usage and reliance upon chatbots for medical information and advice [109], it is essential to address and minimize the risk of misinformation produced by chatbots. One of the most well-known risks associated with chatbot use includes hallucinations, which occur when a generative AI outputs information such as a source or a fact that does not exist [110]. Another problematic aspect of information and recommendations generated by many chatbots is sycophancy, a pattern of responses that likely results from the prioritization of positive human feedback. Sycophancy in chatbots leads to affirmation of the user’s preconceived biases regardless of their truthfulness through behaviors such as rescinding factual information that contradicts the user’s beliefs and producing false outputs that are aligned with the user’s desires [111]. There is also the possibility of replication and amplification of biases in training data, which can lead to the perpetuation of misinformation and healthcare disparities [29]. It is essential to minimize misinformation to ensure that chatbot-enhanced healthcare is as ethical and evidence-based as possible to minimize harm and injustice [112]. Further study to understand the cause of these errors is warranted. Potential solutions include evaluating and adjusting training data for biases and the development of training methods and feedback that prioritize truthfulness over user ratings [113,114]. Table 1 summarizes the latest studies.

**Table 1:** Selected studies evaluating AI chatbots and LLM-based conversational tools relevant to bowel tumor care Note: The studies summarized in Table 1 are heterogeneous and include patient-facing interventions, mixed gastrointestinal oncology cohorts, clinician-facing decision-support evaluations, and simulated vignette or image-based tasks. Reported metrics should therefore be interpreted within each study’s clinical role, design, comparator, and outcome definition rather than as directly comparable efficacy estimates. CRC: colorectal cancer, PN: peripheral neuropathy, LOA: loss of appetite, QoL: quality of life, QLQ: Quality of Life Questionnaire, SUS: System Usability Scale, SCC: squamous cell carcinoma, ADC: adenocarcinoma, MDT: multidisciplinary team, ASA: American Society of Anesthesiologists, AI: artificial intelligence, HCPs: healthcare professionals, NCCN: National Comprehensive Cancer Network, LLM: large language models, EHRs: electronic health records, TBD: to be determined

Study ID	Country	Sample	Demography	Chatbot(s) or LLM(s) used	Pathology studied	Duration of study	Measured outcomes	Results	Limitations	Recommendations
Albino de Queiroz et al. 2023 [40]	Brazil	30 (13 intervention, 17 control)	Mean age 50.3; 43.3% female, 56.7% male	Chatbot developed by researchers using Dialogflow software	CRC*	8 weeks	Symptoms and side effects; Physical activity; Food; QoL* (QLQ*-30 and QLQ-CR29 questionnaires for cancer and colorectal patients, respectively); Frequency of use	Symptoms and side effects: 4 weeks out of 8 on average, with symptoms reported, with 1 participant out of 30 not reporting any. The intervention group reported symptoms such as fatigue, pain, PN*, and LOA* more frequently and accurately than the control group. Physical activity: 5 out of 13 participants reported performing physical activity before diagnosis. During the intervention, all patients were active for at least 1 week, and 7 patients out of 13 were physically active for most of the weeks of their participation. After the intervention, 5 were regularly physically active, and 2 were occasionally active. Participants in the control group who reported physical activity dropped from 11 to 5 out of 17 after treatment. These results suggest that the chatbot and wearable encouraged exercise. Food: 9 out of 13 participants in the intervention group reported changes in food intake. 6 reports described a decrease, 2 described no change, and 2 described an increase (1 person reported twice). QoL: 6 of 13 people in the intervention group completed both questionnaires. Frequency of use: Mean use time: 4 out of 8 weeks. Most patients considered the intervention model efficient, enjoyable, secure, pleasant, and innovative. The mean SUS* survey score was 79.6, with an acceptable usability score being 68.	Despite an even split between male and female patients in the sample, the intervention group consisted mostly of women, while the control group consisted mostly of men. Small sample size.	N/A
Bresler et al. 2025 [115]	United States of America	270 questions	N/A	ChatGPT-4: Gemini 1.0 Ultra	Anal SCC*, small bowel, ampullary, and pancreatic ADC*, biliary tract, and gastric cancers	N/A	Accuracy: Correctness	ChatGPT-4: Correctness: 77.78%, Accuracy: 64.81%. Gemini 1.0 Ultra: Correctness: 75.93%, Accuracy: 57.41%. ChatGPT-4 versus Gemini 1.0 Ultra score differences were not statistically significant.	Accuracy levels fall below the acceptable threshold for clinical use.	N/A
Chatziisaak et al. 2025 [68]	Switzerland	98 (MDT* 1, pre-therapeutic; 94 (MDT 2, post-therapeutic)	Mean age 70; 58% male 42% female	ChatGPT-4	CRC	4 months	Concordance between therapeutic recommendations generated by ChatGPT-4o and those proposed by an MDT based on German S3 and internal hospital guidelines.	MDT 1 (Pre-therapeutic, n=98): Complete concordance: 72.5% (71 patients); Partial concordance: 10.2% (10 patients); Discordance: 17.3% (17 patients). MDT 2 (Post-therapeutic, n=94): Complete concordance: 82.8% (77 patients), Partial concordance: 11.8% (11 patients), Discordance: 5.4% (5 patients); MDT 1 had a significantly higher discordance than MDT 2. MDT was found to be more in agreement with the German S3 guidelines than ChatGPT-4o. Discordance was associated with an ASA score of III or IV and an age of 77 or greater. Partial discordance was associated with rectal cancer and N1 nodal status.	Chatbot cannot account for factors like frailty, functional reserve, or psychosocial determinants. ChatGPT cannot use visual input, such as imaging, to make decisions. ChatGPT categorized patients above 77 years of age and ASA* ≥ III as palliative care cases.	N/A
Gomaa et al. 2023 [42]	United States of America	27	Mean age: 60.97 15 (44%) male, 19 (56%) female	Chatbot made by researchers as part of the Real-time Chemotherapy-Associated Side Effects Monitoring Supportive System (RT-CAMSS)	Pancreatic cancer, CRC, Gastric or esophageal cancer	2 months	Feasibility; Acceptability; QoL; Patient activation; Symptom distress	Feasibility is determined by the criterion of an accrual rate exceeding 65% and attrition below 30%. 68% consent rate, 20.6% attrition rate. Surveys indicate high levels of intervention acceptability. Usability of the intervention received a mean score of 4.47, while the helpfulness of the intervention received a mean score of 3.73 on a scale of 1-5. QoL was assessed by conducting and analyzing 9 interviews, which revealed that the intervention gave them empowerment, self-regulatory skills, and emotional support. Accessible and adaptable information about their symptoms enhanced understanding, communication, and decision-making. Symptom distress was determined by the chatbot symptom checker and multiple scales and assessment tools, such as variants of the FACTs (Functional Assessment of Cancer Therapy). 27 (79%) patients used the chatbot system checker at least once. Symptoms were predominantly fatigue, PN, change in taste, LOA, and constipation. Patient Activation Measure (PAM) results indicated that patients who engaged in texting back for additional information through the system displayed a noteworthy improvement in PAM.	Small sample size. Decreased use post-intervention due to reasons such as a desire to avoid cancer reminders and a lack of symptoms to report. No control group. Chatbot lacks National Language Processing (NLP).	N/A
Horesh et al. 2025 [116]	United States of America	15	Mean age 66.5 years; 46.5% male, 53.5% female	ChatGPT	Rectal cancer	N/A	Concordance between ChatGPT recommendations and MDT decisions was assessed on a scale from 1-5. Justifications were evaluated by 3 experienced colorectal surgeons.	ChatGPT achieved a high concordance rate with MDT decisions (average 4.08 out of 5). Interrater agreement on concordance was found to be moderate. Agreement on justification was slight.	Small sample size. Retrospective study. Chat-GPT sometimes requires multiple prompts to generate an adequately specialized treatment plan.	N/A
Jo et al. 2024 [63]	South Korea	10	Questions selected from patient surveys and testimonials published on the websites of top-rated hospitals	ChatGPT-4; Google Bard; CLOVA-X	CRC	N/A	Accuracy of information, Completeness of answer, Level of empathy, Humanness, Patient satisfaction with answer, Mean of total score. Each AI* was independently evaluated by an MDT of 5 HCPs* and a panel of 5 patients based on the previous four criteria on a Likert scale of 1-5. An informational book on CRC was used as a control. Means of total scores for CRC book used as a control are as follows: MDT only: 11.8, patients only: 14.1, combined: 13.0	Mean Total Scores (MDT only/Patients only/Combined): ChatGPT-4o: 13.5/15.2/14.4. CLOVA-X: 12.2/14.4/13.3. Google Bard: 11.5/15.5/13.5. CRC Book Control: 11.8/14.1/13.0. ChatGPT-4o scored significantly higher than the print book and Google Bard in the MDT evaluation group.	The CRC information book may be outdated (published in 2020). The CRC information book cannot engage in conversation the way that chatbots can. Small sample size.	N/A
Kaiser et al. 2024 [117]	United States of America	108 responses, 27 prompts	Prompts created by two clinician trainees based on the 2024 NCCN guidelines for the management of colon cancer. Prompts were defined by page number within the guideline and grouped into three subtypes: evaluation, management, and surveillance. After question prompts were generated, they were reviewed and verified by three board-certified clinicians, with their responses being considered as the gold standard.	ChatGPT; Microsoft copilot	CRC	N/A	The CLEAR (complete, accurate, evidence-based, appropriate, and relevant) framework was used to develop the following rubric: 1 - completely incorrect or hallucination; 2- partially correct but contains incorrect or misleading information; 3 - partially correct, but missing information; 4 - completely correct. Three participating clinicians graded each response. The final score for each individual response was the mode of the grades. The accuracy of each LLM* response was judged by comparing it to human expert responses based on the latest evidence at the time.	The mean grade for the 108 responses across 27 prompts was 3.06/4. The responses were graded as completely correct for 36% of responses. 38% of responses were missing information, and the remaining 26% were inaccurate or wrong. There was no significant difference between Chat-GPT and Microsoft Copilot.	The selection of LLMs is limited by availability at a single institution, which provides a limited sample of publicly available LLMs. Pre-training data for models is proprietary and not subject to public scrutiny or evaluation. Queries were based solely on the NCCN guidelines. A variety of guidelines available (e.g., American Society for Clinical Oncology) may cause LLMs to deviate from one guideline. This may not account for the outright incorrect or misleading LLM-generated responses observed in this study. Applications outside of clinical oncology have found these tools to be more accurate	N/A
Lau-Min et al. 2024 [75]	United States of America	40	Median age: 64.5; 52.5% male, 47.5% female	PENNY-GI	Primary site of patients: 35% colon, 20% rectum 15% biliary tract 10% appendix 7.5% stomach 7.5% pancreas (adenocarcinoma) 2.5% PNET 2.5% ampulla	3 months	Proportion of chatbot messaging exchanges with incorrect medication or symptom management recommendations as determined by two independent clinician reviewers; Accuracy of the chatbot's understanding of patient messages; Patient-reported adherence; Patient engagement	284 (7.3%) of 3,895 medication-related text messaging exchanges demonstrated PENNY-GI giving incorrect medication management recommendations. The most common errors concerned timing (52.8%), dosage (31.0%), unscheduled downtime (16.2%). 13 (2.5%) of 527 symptom-related text messaging exchanges demonstrated incorrect symptom management recommendations because the chatbot misinterpreted self-reported symptoms (38.5%) or syntax (38.5%), and lack of automated follow-up (23.1%). Gradual decrease in errors as adjustments were made to PENNY-GI in response to chatbot feedback. No chatbot errors appeared to result in serious patient safety concerns. Accuracy was 98.0%. Adherence was 71.8%. Engagement was 44.2%.	Small sample size. Restriction to patients on capecitabine monotherapy. Misalignments between medication reminders and capecitabine dose changes. Rudimentary symptom management feature. Suboptimal medication adherence and engagement with weekly symptom check-in messages. Intensive study oversight.	Present Digital Health Interventions (DHIs) as one of many tools for patients Robust standards for privacy. Integrate DHIs with EHRs to better coordinate treatment plans Design a versatile DHI platform. Standardized documentation of oral cancer-directed therapy dose adjustments in EHRs* Leverage more powerful LLMs Triangulate multiple data sources when evaluating the effect of DHIs on medication adherence Targeted language in messaging used in DHIs Use DHIs to address simple questions and manage low-grade symptoms Incorporate safeguards such as closed-loop communication to minimize the number of erroneous messages.
Maida et al. 2025 [80]	Italy	10	Mean age 60; 60% male, 40% female	ChatGPT-4o	CRC	N/A	Accuracy, completeness, comprehensibility, whether the answer is complete, understandable, and trustworthy	Five experts rated the responses of ChatGPT over two sessions. Mean scores were 4.1 out of 5 for accuracy, 4.2 for completeness, and 4.3 for comprehensibility. Patients rated responses as complete in 97.5%, understandable in 95%, and trustworthy in 100% of cases. Similarity between responses was 86.8%.	Small sample size. Only one chatbot is used. Interactions conducted solely in Italian.	N/A
Massimi et al. 2025 [34]	Italy	100 polyps from 99 Japanese patients	87% adenoma, 13% non-adenoma. Paris classification: Is: 49, IIa: 34, Isp: 9, Ip: 8 polypoid:nonpolypod 66:34	ChatGPT-4o	Colorectal polyps	N/A	Accuracy compared to expert and non-expert human endoscopists; Sensitivity and specificity compared to expert and non-expert human endoscopists. Interobserver agreement of three independent context-reset runs of GPT-4o compared to the interobserver agreement of three experts and three non-experts	GPT-4o accuracy for differentiating non-polypoid from polypoid lesions was 73%, comparable to experts (75%) and non-experts (77%), with similar sensitivity and specificity. Accuracy for differentiating sessile from pedunculated lesions was 55%, lower than experts (76%) and non-experts (77%), p < 0.05. This may be due to poor specificity (12% vs. experts 82% and non-experts 88%; p < 0.01. Experts showed moderate concordance. Non-experts were slightly lower. Three context-reset chatbot runs showed moderate concordance.	The complexity and subtlety of Paris-based morphological distinctions challenge both human and AI raters. A cohort of 100 polyps warrants external validation with a larger sample size. Risk of type-I error inflation. Semi-pedunculated (Isp) and pedunculated (Ip) lesions were grouped to maximize the stalk-bearing sample size.	N/A
Pellegrino et al. 2024 [55]	Italy	587	N/A	ChatGPT-4o	N/A (Quality of bowel preparation for colonoscopy)	N/A	Degree of agreement between ChatGPT-4o and a human evaluator.	ChatGPT-4 deemed 62.9% of frames to be adequately prepared compared to 89.3% of frames assessed by human assessors. The agreement was slight, and concordance was moderate.	Risk of hallucinations. ChatGPT-4o is better suited for text than image inputs. Uncontrolled evaluation.	N/A
Pereyra et al. 2024 [106]	Argentina	10 multiple-choice questions	5 screening, 5 surveillances	ChatGPT-3.5	CRC	N/A	Number of correctly answered clinical vignettes out of 10 compared to an expert, with and without the help of a validated CRC screening mobile application	The average performance of ChatGPT was 45%. Mean correct answers were 2.75, 1.75, and 4.5 for screening, surveillance, and total questions, respectively. ChatGPT showed inconsistency in 4 questions. 238 physicians also responded to the assessment: 123 (51.7%) without and 115 (48.3%) with the mobile app. The mean number of total correct answers of ChatGPT was significantly lower than those of physicians without (5.62) and with the mobile app (7.71); p < 0.001.	Small sample size. Risk of hallucinations. ChatGPT-3.5 may give multiple different answers when presented with the same question more than once.	N/A
Rau et al. 2024 [54]	Germany	50 cases	Mean age 50; 56% female, 44% male	Gastrointestinal imaging-aware chatbot, or GIA-CB (modified from ChatGPT-4 with training); ChatGPT-4	17 GI malignancies, 15 inflammatory disorders, 8 obstructive disorders, 6 benign neoplasms, 2 vascular pathologies, 2 miscellaneous conditions	N/A	Rate of main diagnosis being matched with ground truth; The rate of ground truth being among the top differential diagnoses; Rate of at least one ground truth differential diagnosis matching the chatbots' differential diagnoses; Mean response time; Mean cost per case prompt	GIA-CB (Specialized): Matched the main ground diagnosis for 39/50 cases (78%). Listed the main ground truth diagnosis among its top differentials for 45/50 cases (90%). Contained at least one appropriate diagnosis among its differentials in 49/50 cases (98%). Median response time: 29.8 s, mean cost per case was $0.15 Standard ChatGPT-4: Matched the main ground truth diagnoses 27/50 times (54%). Listed the main ground truth diagnosis among its top differentials for 37/50 cases (74%). Contained at least one appropriate diagnosis among its differentials in 46/50 cases (92%), median response time:15.7 s, mean cost per case: $0.02	An insufficient knowledge retrieval mechanism will lead to incorrect responses, which the AI will treat as being correct due to missing context. Rare diseases may not be identifiable through this approach. Generalizability and appropriateness for real-world data TBD*.	N/A
Siu et al. 2025 [64]	Australia	8 questions used to generate 8 responses	N/A	ChatGPT-4	CRC	N/A	Evaluation of generated responses by eight colorectal surgeons; Accuracy (1-5), Safety (1-5), Appropriateness (1-5), Actionability (1-5), Effectiveness (1-5), Questionnaires used: Patient Education Materials Assessment Tool (PEMAT-AI) DISCERN (DISCERN-AI), Global Quality Score (GQS), Flesch Reading Ease (FRE), Gunning Fog Index (GFI).	Surgeon Ratings (Medians): Safety: 4.25/5; Accuracy, Appropriateness, Actionability, and Effectiveness: all 4.00/5. Tool Scores: PEMAT-AI: 71.43 ('very good'); DISCERN-AI: 12.00 ('fair'); GQS: 4.00 ('high'). Readability: Poor/difficult for general patients (FRE 47.00, GFI 12.40).	ChatGPT-4's responses were evaluated to be inaccessible to patients with less access to education. Limited generalizability due to small sample size and the evaluating sample population being much more expert in CRC than the general population.	N/A
Zeng et al. 2025 [51]	Canada; Italy; Romania; United Kingdom	54 artificial cases	N/A	ChatGPT-4.5	Colorectal neoplasms	N/A	Responses classified as “Correct,” “Partially Correct,” or “Incorrect” based on clinical guidelines and expert recommendations for each country	Canada: Correct responses: 50.0% (27/54) of cases. Partially correct responses: 42.6% (23/54); Incorrect responses: 7.4% (4/54). Italy: Correct responses: 63.0% (34/54) of cases in Italy. Partially correct responses: 27.7% (15/54). Incorrect responses: 9.3% (5/54). Queries in Italian yielded correct guidance for 64.8% (35/54) of cases. Italy lacked detailed guidelines for polyp management and post-test surveillance. Romania: Correct responses: 40.7% (22/54) of cases in Romania. Partially correct responses: 59.3% (32/54). Incorrect responses: 0% (0/54). Romanian queries were accurate for 40.7% (22/54) of cases. Romania lacked detailed guidelines for polyp management and post-test surveillance. United Kingdom: Correct responses: 55.6% (30/54) of cases in the United Kingdom. Partially correct responses: 31.5% (17/54), Incorrect responses: 12.9% (7/54)	Inconsistency between ChatGPT-generated titles and corresponding recommendations may impact its reliability in clinical decision-making. Lack of transparency regarding ChatGPT’s training data. The dataset is biased towards the English language. There is a difference in performance between the free and paid versions of ChatGPT. Responses heavily depend on the training dataset and may not reflect local practice. Need for standardization and integration with EHRs and clinical practice.	N/A
Zhou et al. 2024 [56]	China	150 questions	N/A	8 models: ChatGPT-3.5; ChatGPT-4; ChatGPT-4 Turbo; Google Bard; Claude 2.1; LLaMA-2-70B; Doctor GPT; Mixtral-8x7B	CRC	21 weeks	Nine oncologists (three residents, three fellows, and three attendings) answered the same questions as the chatbots. Answers provided by the eight chatbots and nine doctors were evaluated by the authors. Answers were scored as 1 or 0 based on whether they followed the NCCN guidelines. The total score for each question was determined by the number of answers aligned with the guidelines (0 – 3). Accuracy calculated as the number of correct answers out of 150.	Accuracy Rankings: Claude 2.1: 82.67% (outperformed attendings, fellows, and residents); Doctor GPT: 80.45% (outperformed residents and fellows); ChatGPT-4 Turbo: 78.44% (outperformed residents); ChatGPT-4: 78.00% (outperformed residents); Mixtral-8x7B: 73.33% (outperformed residents); Google Bard: 70.00%; ChatGPT-3.5: 64.89%; LLaMA-2-70B: 61.78%.	Small sample size. Limited generalizability. Closed-ended questions. Subjective scoring for accuracy. Chatbots are not trained with medical data.	N/A

Ethical and safety considerations

Evidence evaluating AI chatbots and LLMs in CRC has reported variable performance across different clinical tasks and outcome measures, including screening, diagnosis, treatment recommendations, prognosis, guideline concordance, and the appropriateness or scientific adequacy of responses [56,64,118-121]. These measures should not be interpreted as a single, directly comparable measure of “accuracy,” and reported performance varies substantially according to the clinical task, question format, evaluation criteria, and model assessed. Several studies have reported performance below 80%, while others have demonstrated higher levels of task-specific agreement or accuracy. Overall, the available evidence suggests that chatbot performance may decline when questions require greater clinical complexity, contextual interpretation, or nuanced decision-making, indicating that these systems should not currently be considered equivalent to expert clinical judgment [56,64,118-121]. For example, ChatGPT has demonstrated better performance on direct questions than on more nuanced questions concerning colonoscopy surveillance intervals based on American Gastroenterological Association guidelines. However, these findings should be interpreted in light of methodological limitations, including potential human adjudication error, the small number of assessors, and variability in evaluation related to the formulation and length of chatbot responses [122].

Chatbots pose a security concern if not used correctly. Their integration into healthcare could require the collection and storage of protected health information, which raises concerns for data security and privacy [123]. When using the tool to perform a task, users may inadvertently provide sensitive personal information to the public domain. These sensitive pieces of information can be disclosed to the intended and unintended audience [123]. According to their privacy policy, they can share information without notifying the owner of that information, unless required by law [124]. The use of AI chatbots in healthcare should likely be used in compliance with the Health Insurance Portability and Accountability Act (HIPAA), ensuring confidentiality, integrity, and availability of protected health information as it is collected, stored, and shared [123].

In a study conducted by Stuij et al., in which a digital communication skills training tool was designed to provide information for oncologists’ learning, it was found that some participants felt hesitant to provide information due to feeling vulnerable [125]. In a study that addresses privacy concerns for interaction with AI chatbots, 13 participants interacted with AI chatbots to identify privacy harms and risks of using AI chatbots [126]. Addressing these harms and risks is vital to encourage interaction with AI chatbots. This can be done by familiarizing users, training them on what sensitive information can and cannot be shared, clear and transparent and clear privacy guidelines [126].

The rapid evolution of AI in healthcare has led to the emergence of tools that lack regulatory approvals and pose ethical and legal concerns [127]. In June 2021, the WHO issued indications, recommendations, and guidelines about the development, application, and utilization of AI in medicine. Principles and guidelines include autonomy protection, promoting responsiveness and sustainability, promoting accountability and responsibility, promoting welfare and safety, ensuring transparency and explainability, and ensuring inclusivity and equity [128].

Implementation challenges

Internet access, device availability, bandwidth, data costs, server requirements, and reliable electricity differ vastly across LMICs and may limit chatbot deployment [90]. Connectivity should therefore be viewed as a context-specific implementation constraint rather than a single uniform percentage across LMICs. Offline or low-bandwidth models and public-private infrastructure partnerships may enhance feasibility, but implementation requires a local assessment of access, affordability, maintenance, and technical support [90,129].

While many AI chatbots can support multiple languages, training data are still overwhelmingly English-language, and performance may be worse in under-represented languages [130]. LMICs also have significant cultural, social, and religious diversity that may not be sufficiently represented in model training. Therefore, patient- and population-centered development, local-language validation, and culturally informed governance are important prerequisites for context-appropriate implementation [90,131].

Future directions

Trust is one of the central features of the patient-physician relationship [132]. A high level of trust is linked to beneficial health behavior, fewer symptoms, and a better quality of life [133]. There are no studies assessing how much trust patients would place in AI chatbots, or whether they can have the same effective level of trust that is usually built between a patient and their treating physician, while providing the same beneficial outcomes.

There is an increasing use of AI chatbots in cancer prevention, education, screening, management, and surveillance [134]. Electronic medical records, which store medical processes like patient reception, examination, blood tests, medication, surgery, and medical expenses, are evaluated as the most reliable data in the healthcare system [135,136]. Using electronic medical records as a data source for AI is crucial [137].

Specialized AI systems and LLM-based chatbots are considered task-specific and may improve performance in selected diagnostic, educational, documentation, or decision-support tasks [8]. Hybrid human-AI approaches have also been proposed for emergency triage and resource prioritization, although these frameworks often involve specialized predictive systems rather than patient-facing chatbots [138]. Future studies should therefore evaluate clearly defined clinical tasks, compare human-only, AI-only, and human-AI workflows, and prioritize safety, equity, and prospective real-world validation.

## Conclusions

AI chatbots have meaningful potential to complement bowel tumor care through patient education, symptom monitoring, communication, and selected decision-support functions, particularly where healthcare resources are constrained. However, the current literature is heterogeneous, dominated by studies from high-income settings, and does not establish broad clinical effectiveness, safety, cost-effectiveness, or equity benefits in LMICs. Chatbots should therefore be implemented as clinician-supervised adjuncts rather than autonomous substitutes for professional care. Prospective validation in diverse LMIC settings, robust privacy and governance safeguards, local-language and cultural adaptation, and careful evaluation of digital access and workflow integration will be essential before wider adoption.
